# Overqualification at work and risk of hospitalization for psychiatric and somatic diseases among immigrants in Sweden – a prospective register-based study

**DOI:** 10.5271/sjweh.4055

**Published:** 2022-10-29

**Authors:** Maria Brendler-Lindqvist, Martin Tondel, Magnus Helgesson, Tobias Nordqvist, Magnus Svartengren

**Affiliations:** 1 Department of Medical Sciences, Occupational and Environmental Medicine, Uppsala University, Uppsala, Sweden.; 2 Occupational and Environmental Medicine, Uppsala University Hospital, Uppsala, Sweden.; 3 Department of Clinical Neuroscience, Division of Insurance Medicine, Karolinska Institutet, Stockholm, Sweden.

**Keywords:** discrimination, emigration, employment, immigration, labor migrant, migrant worker, mismatch, occupational exposure, occupational health, refugee, status incongruence, status incongruency, status inconsistency, occupation, working condition, work exposure, work-related health

## Abstract

**Objectives:**

This study aimed to (i) describe the prevalence of overqualification at work among immigrants in Sweden and (ii) analyze any association between overqualification and the risk of hospitalization for somatic and psychiatric disease among refugees and labor immigrants.

**Methods:**

We performed a prospective register study in a cohort of 120 339 adults who immigrated to Sweden in 1991–2005 and were employed in 2006. Education-occupation status was defined as the combination of an individual’s highest level of education and their occupation skill level. Individuals were followed from 2007 to 2016 with regard to hospitalization for a psychiatric, cardiovascular, respiratory or musculoskeletal disease or diabetes. Hazard ratios (HR) with 95% confidence intervals (CI) were calculated in a multivariate Cox regression analysis adjusted for age, gender, reason for residence and duration of residence.

**Results:**

The overall prevalence of overqualification among immigrants with an academic education was 39%. Overqualified individuals had an increased risk of hospitalization for any disease (HR 1.33, 95% CI 1.21–1.46) compared to “job-matched with an academic education”. However, the risk estimates were lower than that of “job-matched with no academic education” (HR 1.56, 1.46–1.68). The increased risk of hospitalization for a psychiatric disease of overqualified individuals did not differ from that of job-matched with no academic education.

**Conclusion:**

Our study showed that being overqualified was associated with poorer health outcomes than job-matched individuals with an academic education. Considering the high prevalence of overqualification in immigrants, this constitutes a concern, for both society and individuals.

Migration is a growing phenomenon, and in 2019 the number of international immigrants reached almost 272 million globally (1). In Sweden, immigrants made up about 25% of the working age population in 2020, a proportion that is expected to increase (2). In recent years, about half of newly arrived immigrants had a post-secondary education, a figure higher than among the Swedish-born population (3). Immigrants make important contributions to the Swedish labor force, but labor market integration of immigrants also brings challenges. Studies have shown that immigrants have higher rates of un- or underemployment, overqualification, and allocation to more physically demanding and lower paid occupations than the non-immigrant population (4, 5).

One adverse working condition that affects immigrant populations to a high extent is overqualification, ie, a situation where the educational or skill attainment exceeds the qualifications required for the occupation. The rate of overqualification among immigrants with post-secondary education was 10% points higher than among the non-immigrant population in the OECD countries in 2017 (6). In Sweden, recent numbers have shown that 74% of immigrants with an academic education were employed in an occupation that matched their qualifications, compared with 90% among the Swedish-born (7). The health consequences of overqualification are not fully understood. While some studies found negative effects of overqualification on self-rated health (8, 9), mental health (10, 11), work injuries (12), incidence of cardiovascular disease (13) and mortality (14), there have also been studies not finding any such associations (15–17). One possible explanation for the observed negative health effects is that overqualification leads to psychological stress due to a sense of inferiority and lost social status (14). Over time, such stress may increase the risk of cardiovascular disease and diabetes through biological stress response mechanisms and maladaptive coping behaviors, such as alcohol consumption and smoking (14, 18). Negative health effects may also be due to social and organizational factors at the workplace, for example overqualified individuals may suffer from lack of relevant vocational training for the job and less solidarity from colleagues increasing the risk of exposure to work hazards (12).

Despite the magnitude of the problem with overqualification among immigrant workers, only four studies were identified that focused specifically on immigrant populations (8, 10, 11, 19). These studies were small, survey-based, and focused on selected groups, such as newly arrived immigrants. Moreover, most of the studies made use of cross-sectional analysis, which limits the possibility of drawing conclusions regarding causal directions. Thus, there is a need to better understand the long-term health consequences for this particular group. To the best of our knowledge, there is no former study on the health consequences of overqualification among immigrant populations with a prospective study design using objective measurements of health status, such as hospitalization for somatic and psychiatric diseases. Most immigrants to Sweden are refugees, who make up a more vulnerable group when it comes to both health and the risk of overqualification. By studying a large, economically active population of immigrants to Sweden – including refugees and labor immigrants – we were able to increase knowledge on how overqualification affects the health.

The aims of this study were to describe the prevalence of overqualification among immigrants in Sweden and to analyse any association between overqualification and the risk of hospitalization for somatic and psychiatric disease among refugees and labor immigrants.

## Methods

### Study design

This was a prospective cohort study based on register data. The index population was defined on 31 December 2006 and included all individuals born in 1942–1987 who immigrated to Sweden from selected geographical regions in 1991–2005 and who were aged 18–59 years at the time of immigration (N=287 635). The selected regions were: (i) Eastern Europe, Russia and the post-Soviet republics, (ii) the West (including Western Europe, USA, Canada, Australia and New Zealand), (iii) the Middle East, (iv) the Horn of Africa and Sudan, (v) South and Central America, (vi) East Asia and (vii) other. These regions are the origin of the vast majority of non-Nordic immigrants to Sweden and were defined in order to create somewhat homogenous units in terms of national income level, language, culture and religion. As 65 years was the regular retirement age in Sweden in 2006, individuals aged >64 years at baseline were excluded. Only individuals with a registered income from employment in November 2006 were included. In the index population, 48% (N=137 947) did not fulfil this criterion. Moreover, immigrants were excluded from the study if there was information on neither reason for residence (N=1307) nor education and/or occupation (N=27 213). We also excluded individuals hospitalized in 2006 for a psychiatric, cardiovascular, respiratory or musculoskeletal disease or for diabetes, as these are diseases of a persistent nature or with a high risk of relapse, which can be assumed to affect work ability (N=829). The final study population consisted of 120 339 individuals, who were followed from 1 January 2007 to 31 December 2016. The study population differed from the index population mainly regarding time since residence as 25% of the included individuals had been in Sweden less than six years, compared to 52% among those who did not meet the inclusion criteria.

All data were retrieved from national registers at Statistics Sweden and the Swedish National Board of Health and Welfare and linked at an individual level through the unique personal identification number assigned to each individual when obtaining a residence permit in Sweden. These numbers were replaced by a serial number by Statistics Sweden before data were made available to the researchers in order to guarantee anonymity. Information on demographic characteristics and reason for residence were retrieved from Statistics Sweden’s longitudinal database for integration studies (STATIV). Immigrants were defined as individuals born outside Sweden with two parents born outside Sweden. Immigrants from the Nordic countries were not included in the study as information on reason for residence is largely lacking in the registers due to an agreement on free movement between the Nordic countries.

The Regional Ethics Review Board in Uppsala, Sweden, approved the study (file number 2021-01893).

### Variables

*Exposure*. In this study, education-occupation status was defined in an objective way by combining information on an individual’s education and occupation from the longitudinal integrated database for health insurance and labor market studies (LISA). Information on individuals’ occupation titles is provided by employers on a yearly basis and classified based on the Swedish Standard Classifications of Occupations (SSYK96). SSYK is a national adaptation to the International Standard Classification of Occupations (ISCO-88) and is a hierarchical classification with four levels, based on the type of work performed and the skill level required. The baseline year of 2006 was chosen as this was the first year when information on occupation was available for all sectors. Information on education obtained in Sweden was retrieved from education registers. Information on foreign education was retrieved from multiple sources. From 1999 and onward, each immigrant receives a survey from Statistics Sweden in the year after obtaining residency with questions regarding education level. Supplementary surveys to immigrants for whom there is no information on education in the register were sent out in 1995, 1999 and 2005/2006. Survey information is updated later, when immigrants register their qualifications in contacts with the Swedish authorities. For the purpose of this study, academic education was defined with a cut-off of post-secondary education of three years or more. Managerial work, occupations requiring an advanced level of higher education and occupations requiring higher education qualifications or the equivalent were defined as qualified occupations for an individual with an academic education. All other occupations were defined as non-qualified. Thus, overqualification was defined as having a post-secondary education of ≥3 years and an occupation not requiring higher education qualification according to SSYK96. Conversely, an individual with <3 years of post-secondary education and an occupation requiring higher education was defined as underqualified. Individuals with post-secondary education of ≥3 years and an occupation requiring higher education were defined as “job-matched with an academic education”, while individuals without post-secondary education and an occupation not requiring higher education were defined as “job-matched with no academic education”.

### Outcome

Data on main discharge diagnoses from in-patient care at Swedish hospitals coded with the International Classification of Diseases, version 10 (ICD-10) (20) were retrieved from the National Patient Register (NPR). NPR is held by the Swedish National Board of Health and Welfare, and the reporting of data is mandatory for the County Councils. The outcome was defined as being hospitalized at least once for selected diagnoses from the ICD-10 chapters mental and behavioral disorders, diseases of the circulatory system, diseases of the respiratory system or diseases of the musculoskeletal system and connective tissue, or having a diagnosis of diabetes, hereafter referred to as psychiatric, cardiovascular, respiratory and musculoskeletal diseases (table 1). The selected diagnoses represented 81% of all hospitalizations in 2006 and are diseases of a persistent nature or with a high risk of relapse.

**Table 1:** Cumulative incidence rate (%) of hospitalization by disease group in the study population between 2007 and 2016, stratified by educationoccupation status (N=120 339). [ICD-10=International Classification of Diseases, 10^th^ revision.]

Diagnoses according to ICD-10	Job-matched with academic education		Overqualified		Underqualified		Job-matched with no academic education	
			
	Men N=10 966	Women N=12 143	Men N=7140	Women N=7807	Men N=3660	Women N=3183	Men N=38 125	Women N=37 315
							
	%	%	%	%	%	%	%	%
Diabetes (E10–E14)	0.1	0.0	0.1	0.0	0.2	0.1	0.2	0.1
Psychiatric (F31–F38, F41, F43, F45, F48)	0.8	1.2	1.3	1.9	1.2	2.0	1.5	2.1
Cardiovascular (I10–I13, I20–I25, I50, I60–I69)	2.3	0.8	3.8	1.3	2.9	1.3	3.8	1.4
Respiratory (J13–J18, J20, J40–J45, J60–J69, J92)	0.6	0.6	1.0	0.7	1.0	0.5	1.1	1.0
Musculoskeletal (M05–M06, M15–M17, M50–M54, M65, M70–M71, M77–M79)	1.3	1.4	2.1	2.0	2.0	3.0	2.4	2.8
Any of the selected diagnoses	4.8	3.9	7.9	5.6	6.7	6.3	8.4	7.0

### Covariates

Demographic characteristics considered in the statistical model were sex, age at baseline, duration of residence and reason for residence. Age at baseline was derived as the difference between 2006 and year of birth and presented in four age groups: 19–29, 30–39, 40–49 and 50–64 years. Duration of residence was calculated as the difference between 2006 and year of residency registered by the Swedish Migration Agency, and was categorized as 11–15, 6–10 or 1–5 years. Individuals were classified as (i) refugees, (ii) family reunification immigrants, (iii) labor immigrants, and (iv) others, based on reason for residence as registered by the Swedish Migration Agency. For individuals who were granted residence more than once, information on the last reason for residence was used. The group ‘others’ consisted of 80% of students.

### Statistical analyses

Hazard ratios (HR) with 95% confidence intervals (CI) were calculated with multivariate Cox regression analysis in SPSS version 27 (IBM Corp, Armonk, NY, USA), for hospitalization for any psychiatric, cardiovascular, respiratory or musculoskeletal disease, or diabetes, depending on which occurred first. HR were also calculated for each of the psychiatric, cardiovascular, respiratory, and musculoskeletal disease groups separately. No separate analysis was carried out regarding hospitalization for diabetes due to too few cases. “Job-matched with academic education” was chosen as the reference. Person-years under risk for disease were calculated from 1 January 2007 to the first event of emigration, death or hospitalization for any of the diseases studied, or to the end of study on 31 December 2016. Analyses were adjusted for sex, age at baseline (continuous), reason for residence and duration of residence. Reason for residence and region of origin were highly correlated and therefore could not be included in the same model. We chose to include reason for residence, rather than geographical region, based on the assumption that reason for residence was the more decisive factor for overqualification as the conditions for establishing oneself on the labor market vary considerably between voluntary and forced immigrants. As a sensitivity analysis, we ran a separate model adjusted for region of origin, instead of reason for residence. In order to investigate whether the reason for residence modified the effect of overqualification on hospitalization, we conducted a separate regression analysis restricted to refugees and labor immigrants with an academic education. We then calculated the relative excess risk due to interaction (RERI) of the combination of refugee status and overqualification, as suggested by Rothman for investigation of additive interaction in epidemiological research (21). We also calculated RERI for each sex, as well as for duration of residence (11–15 versus 1–5 years), on the association between overqualification and health among immigrants with an academic education. We used the Delta method to obtain 95% CI for the estimate of the interaction (22). In order to study how a prolonged exposure to overqualification affected the association with health, we ran a separate model including only those individuals (N=77 613) who had a constant education-occupation status during the follow-up period. To estimate the effect of prior health on the association between education-occupation status and health we carried out a sensitivity analysis where we excluded all individuals hospitalized for any of the diseases under study during the period between receiving a residence permit in Sweden and baseline.

### Results

**Figure 1:**
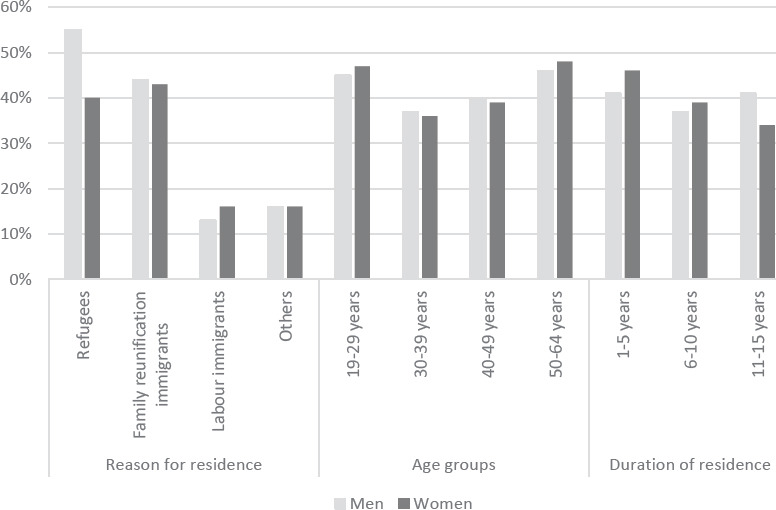
Prevalence of overqualification among immigrants with an academic education by reason for residence, age group and duration of residence at baseline in 2006 (N=38 056).

Refugees and family reunion immigrants constituted 40% and 52% of the study population, respectively, while labor immigrants made up 6.4%. Eastern Europe, Russia and the post-Soviet republics was the most common region of origin, followed by the Middle East and the West. Three quarters of the population were aged 30–49 years, ie, in the middle of their working career, and 47.5% had resided in Sweden for >10 years (table 2). The overall rate of overqualification among individuals with an academic education was 39% independent of sex. The prevalence of overqualification differed considerably depending on the reason for residence. Among males, 55% of refugees with an academic education were overqualified, while the rate was 13% among labor immigrants. Among females, the rate of overqualification was 40% among refugees and 16% among labor immigrants, respectively. Overqualification was more common in the youngest and oldest age groups. Sex differences were generally small, except among refugees where males had the highest rate of overqualification. There was a reduction in overqualification by duration of residence among female immigrants, but not among males (figure 1). The cumulative incidence rate of any of the diseases under study during follow-up was 6.9% in the total population. The cumulative incidence rate for hospitalization for a cardiovascular disease was higher among males than females, while the opposite was true for psychiatric disease (table 1).

**Table 2:** Characteristics of the study population at baseline in 2006, stratified by reason for residence (N=120 339).

	Total N=120 339	Refugees N=47 637	>Family reunification immigrants N=62 098	Labor immigrants N=7722	Others N=2846
				
	%	%	%	%	%
Sex					
Male	49.8	58.3	40.3	68.0	63.5
Female	50.2	41.7	59.7	32.0	36.5
Region of origin					
Eastern Europe, Russia and the post-Soviet republics	46.0	61.1	36.4	36.4	29.2
The Westa	11.5	0.2	14.6	52.0	24.8
Middle East	16.7	24.5	13.2	1.1	5.3
Horn of Africa & Sudan	3.9	5.2	3.4	0.1	1.7
South & Central America	4.5	2.1	6.7	2.1	3.5
East Asia	8.3	1.7	13.2	4.6	23.3
Other	9.0	5.2	12.5	3.5	12.1
Age (years)					
19–29	11.1	4.5	14.7	16.5	29.2
30–39	43.7	35.4	49.4	48.1	46.5
40–49	33.7	43.1	27.9	26.9	20.3
50–64	11.6	17.1	8.1	8.4	4.0
Duration of residence (years)					
11–15	47.5	71.4	35.3	8.3	19.7
6–10	27.7	19.6	34.7	21.9	27.2
1–5	24.7	9.0	30.0	69.8	53.0
Education level					
Academic	31.6	21.1	33.0	69.2	77.0
Non-academic	68.4	78.9	67.0	30.8	23.0
Occupation skill level					
Academic	24.9	15.9	24.3	67.2	72.5
Non-academic	75.1	84.1	75.7	32.8	27.5
Education-occupation status					
Job-matched with academic education	19.2	10.8	18.6	59.4	64.8
Overqualified	12.4	10.3	14.4	9.8	12.2
Underqualified	5.7	5.2	5.7	7.8	7.7
Job-matched with no academic education	62.7	73.7	61.3	23.0	15.3

^a^Including Western Europe, USA, Canada, Australia and New Zealand.

**Figure 2:**
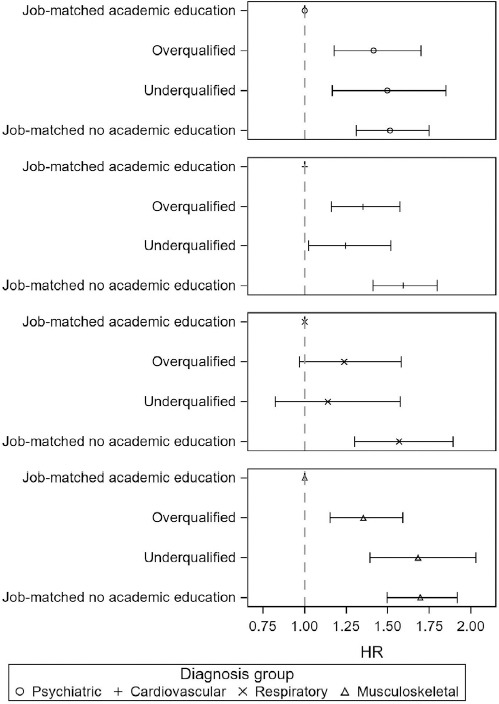
Hazard ratios (HR) with 95% confidence intervals (CI) of hospitalization for each of the selected disease groups by education-occupation status. Adjusted for sex, age (continuous), reason for residence and duration of residence at baseline in 2006 (N=120 339).

Overqualified individuals had a significantly higher risk of being hospitalized for any of the diseases under study than job-matched with an academic education with HR 1.33 (95% CI 1.21–1.46) (table 3). For underqualified individuals the HR was 1.36 (95% CI 1.21–1.46). Job-matched with no academic education had the highest risk of hospitalization for any of the diseases under study with HR 1.56 (95% CI 1.46–1.68). Separate analyses of the risk of hospitalization by disease group showed that over- and underqualified individuals had a significantly increased risk of hospitalization for a psychiatric, cardiovascular or musculoskeletal disease compared with job matched with an academic education (figure 2). The risk estimates of hospitalization for a respiratory disease were increased for over- and underqualified individuals compared with job-matched with an academic education, but the difference did not reach statistical significance. The HR of hospitalization for a psychiatric disease were 1.41 (95% CI 1.18–1.70) for overqualified and 1.50 (95% CI 1.31–1.75) for underqualified and did not differ from the HR of job-matched with no academic education which was 1.51 (95% CI 1.31–1.75). Adjusting for covariates only slightly attenuated the results. The adjustment for region of origin instead of reason for residence did not change the results (data not shown).

**Table 3:** Crude and adjusted hazard ratios (HR) and 95% confidence intervals (CI) for hospitalization for any of the selected psychiatric, cardiovascular, respiratory or musculoskeletal diagnosis, or diabetes by education-occupation status (N=120 339).

	≥1 hospitalizations (2007–2016)	Crude	Mutually adjusted ^a^
		
	N	HR (95% CI)	HR (95% CI)
Education-occupation status			
Job matched with academic education	955	1	1
Overqualified	946	1.42 (1.30–1.56)	1.33 (1.21–1.46)
Underqualified	416	1.43 (1.27-1.60)	1.36 (1.21–1.52)
Job-matched with no academic education	5439	1.71 (1.59–1.83)	1.56 (1.46–1.68)
Sex			
Male	4236	1	1
Female	3574	0.83 (0.79–0.86)	0.85 (0.81–0.89)
Reason for residence			
Refugees	4053	1	1
Family reunification immigrants	3408	0.80 (0.76–0.84)	0.84 (0.80–0.89)
Labor immigrants	268	0.56 (0.50–0.63)	0.65 (0.57–0.74)
Others	81	0.55 (0.44–0.68)	0.67 (0.54–0.84)
Duration of residence in 2006 (years)			
11–15	4461	1	1
6–10	2008	0.99 (0.94–1.04)	1.07 (1.01–1.13)
1–5	1341	0.94 (0.88–1.00)	1.09 (1.01–1.17)

a All models adjusted for age in 2006 (continuous).

Testing for interactions did not show any modifying effect of being a refugee or a labor immigrant on the risk of hospitalization for any one disease under study depending on education-occupation status. Interaction was not seen between overqualification and either sex or duration of residence (1–5 versus 11–15 years) in relation to the risk of hospitalization for any of the diseases under study. In our cohort of immigrants, 64.5% did not change their education-occupation status during follow-up, and among overqualified individuals, 52.9% did not change their status during follow-up. The association between over- and underqualified and the risk of hospitalization remained when the analysis was restricted to those individuals who had an unchanged education-occupation status with HR 1.35 (95% CI 1.20–1.52) for overqualified and HR 1.41 (95% CI 1.21–1.63) for underqualified, respectively. The HR among persistent job-matched with no academic education was 1.41 (95% CI 1.30–1.54). Excluding all individuals hospitalized for any of the diseases under study up to nine years before baseline did not change the association between education-occupation status and hospitalization during follow-up (data not shown).

## Discussion

In this study of a large and representative population of economically active immigrants in Sweden, about a third had an academic education. Of those with academic education, 39.4% did not have an occupation requiring higher education. Similar rates of overqualification have been shown in previous studies (7, 16, 23). Our results showed that overqualified individuals had a risk of hospitalization for any of the diseases under study that was higher than that of job-matched with an academic education, but lower than that of job-matched individuals with no academic education. Moreover, overqualified individuals had a significantly increased risk of hospitalization for a psychiatric, cardiovascular or musculoskeletal disease compared with job-matched with academic education. Regarding hospitalization for a psychiatric disease, the increased risk estimates did not differ between the overqualified and the job-matched with no academic education.

There may be several explanations for the health status observed among overqualified individuals in this study. First, overqualified individuals may experience psychological stress due to reasons such as status incongruence and a sense of inferiority, effort–reward imbalance and perceived work-related discrimination with negative effects on mental health (5, 14, 24). Over time, psychosocial stress may also increase the risk of somatic disease through biological mechanisms as well as negative coping behavior such as smoking (14, 18). Second, overqualified individuals may be exposed to occupational hazards common in occupations with lower educational requirements and thus share exposures with non-qualified workers. Occupations defined as non-qualified in this study included manual occupations with a known risk of exposure to inhaled dust and particles, noise, heavy physical labor, as well as other physical and chemical exposures, increasing the risk of occupational lung diseases (25, 26), cardiovascular disease (27), and musculoskeletal diseases such as chronic back pain, rotator cuff diseases and knee arthrosis (28–30). A third explanation may be that social and organizational factors in the work environment result in a higher risk of exposure to work hazards for overqualified compared with job-matched individuals (12). The fact that overqualified individuals in this study had a similar risk of hospitalization for psychiatric disease as job-matched with no academic education, which was not the case for the other disease groups, lends support to a psychological pathway. This is in line with other studies showing an association between overqualification and poor mental health measured through screening instruments validated for detecting risk of psychiatric disorders (11, 31, 32). Regarding the somatic diseases, our results showed that the risk of hospitalization was higher among overqualified workers than among job-matched with an academic education, but lower than among job-matched with no academic education, which may be due to a combination of the above-mentioned factors. Earlier research on the effects of overqualification on cardiovascular diseases has been inconclusive. While one study found an association between overqualification and ischemic heart disease (13), other studies did not find any associations between overqualification and cardiovascular mortality (17), myocardial infarction or stroke (15). The effect of overqualification on respiratory and musculoskeletal diseases has, to the best of our knowledge, not been studied before.

A striking feature in our data was that about half of the immigrants who settled in Sweden between 1991–2005 did not have a registered income from employment in 2006. Research has documented negative health effects of unemployment (33, 34). Thus, being overqualified may be a better alternative from a health perspective than being unemployed. On the other hand, for educated individuals, unemployment may be considered a form of overqualification, irrespective of the educational level, as they are not able to use their skills in any occupation. However, the health consequences of overqualification among the unemployed were beyond the scope of this study.

Suggested reasons for the high prevalence of overqualification among immigrant populations are lack of recognition of foreign credentials and experiences, lack of connections and networks, poor proficiency in the language of the destination country and possible discrimination by employers (9, 11, 35). In our study, refugees and family reunification immigrants were the groups with the highest rates of overqualification, while the rate was lowest among labor immigrants. Non-labor immigrants may differ regarding educational orientation, as labor immigrants may have chosen to migrate due to job opportunities, while the migration decision was driven by other factors for refugees and family reunification immigrants. The presence of discrimination towards individuals from non-dominant ethnicities may be another factor that affects refugees to a higher extent than labor immigrants, as a greater proportion of refugees originated from non-Western countries (36, 37). It has been argued that working in an occupation for which one is overqualified may provide a pathway toward more qualified occupations. However, in our study, 53% of the overqualified participants remained overqualified for 10 years. This is consistent with another study showing a high persistence of overqualification among immigrants, indicating that overqualification may not be a temporary state in this group (36).

In the study population, underqualification was substantially less common than overqualification and was therefore not the focus of this study. The underqualified presented higher risk of hospitalization than job-matched with an academic education, the reasons need to be investigated in other studies with focus on this form for education-occupation mismatch.

### Strengths and limitations

The major strength of our study was the prospective design, with each individual being followed annually for health outcomes during a ten-year period. This design made it possible to study the long-term health consequences of overqualification, which had not been studied in immigrant populations before. In an international comparison, our study was based on highly qualitative national registers with a complete coverage of all registered immigrants in Sweden during the study period. Refugees constituted about 40% of our study population, a group not specifically studied before. Another strength of our study was the use of registered diagnoses after hospitalization, instead of relying on self-reported data. As the Swedish healthcare system is accessible to all residents on equal terms, and as underreporting of data on inpatient care to the national patient register is low, selection bias is considered to be very small. Thus, our study adds knowledge on the health consequences of overqualification for a broad range of severe somatic and psychiatric conditions, which have not been studied before. By including only health conditions requiring hospital treatment, we could reduce bias due to differences in health-seeking behavior between immigrant groups. There is a tendency that less educated individuals do not approach health services to the same extent as more highly educated individuals, but including only severe cases reduced this kind of bias.

The study also suffered from some limitations. First, we lacked information on lifestyle factors or work-related exposures with possible consequences for health. The role of these factors for the associations between overqualification and health outcomes would be of interest for further studies using other methods. Second, we also lacked information on chronic conditions others than when leading to hospitalization. This means we cannot fully exclude the possibility of bias due to health-related selection as the medical conditions we studied can reduce work capacity long before resulting in hospitalization. A sensitivity analysis including only individuals not hospitalized in Sweden prior to baseline due to any of the diseases under study did not change our results. However, this does not exclude the possibility of a health-related selection, especially among the 25% of immigrants who had resided in Sweden for <6 years. A third limitation was the risk of misclassification, as we were not able to consider informal qualifications, such as on-the-job courses or previous work experience. Such misclassification may have diluted the differences between groups, thus leading to an underestimation of the real effect. Lastly, the study did not include undocumented immigrants, a group of particular risk having hazardous work exposures. According to authorities there were between 20–50 000 undocumented immigrants living in Sweden in 2017, but being relatively few in relation to the total immigrant population it would probably not have changed the overall results had they been included in our study (38)

### Concluding remarks

Overqualification is a substantial problem among immigrants with an academic education on the Swedish labor market. Our study showed that being overqualified was associated with poorer health outcomes than among job-matched individuals with an academic education. Considering the high prevalence of overqualification among immigrants, this constitutes a concern for both society and individuals. Further studies are needed to better understand determinants for overqualification and the mechanisms behind the negative health effects.

## References

[1] International Organization for Migration (IOM) (2019). World Migration Report 2020.

[2] Statistics Sweden Arbetskraftsundersökningarna (AKU) [Labor Force Surveys (LFU)] (In Swedish).

[3] Statistics Sweden (2018). Utrikes föddas utbildningsbakgrund 2017 [Educational background 2017 among foreign born persons] (In Swedish).

[4] Ambrosini M, Barone C (2007). Employment and working conditions of migrant workers.

[5] Sterud T, Tynes T, Mehlum IS, Veiersted KB, Bergbom B, Airila A (2018). A systematic review of working conditions and occupational health among immigrants in Europe and Canada. BMC Public Health.

[6] OECD (2017). International Migration Outlook 2017.

[7] Edström J (2015). Sveriges utrikesfödda akademiker [Foreign-born graduates in Sweden] (In Swedish).

[8] Dunlavy AC, Garcy AM, Rostila M (2016). Educational mismatch and health status among foreign-born workers in Sweden. Soc Sci Med.

[9] Smith P, Frank J (2005). When aspirations and achievements don't meet. A longitudinal examination of the differential effect of education and occupational attainment on declines in self-rated health among Canadian labor force participants. Int J Epidemiol.

[10] Chen C, Smith P, Mustard C (2010). The prevalence of over-qualification and its association with health status among occupationally active new immigrants to Canada. Ethn Health.

[11] Reid A (2012). Under-use of migrants'employment skills linked to poorer mental health. Aust N Z J Public Health.

[12] Premji S, Smith PM (2013). Education-to-job mismatch and the risk of work injury. Inj Prev.

[13] Peter R, Gässler H, Geyer S (2007). Socioeconomic status, status inconsistency and risk of ischaemic heart disease:a prospective study among members of a statutory health insurance company. J Epidemiol Community Health.

[14] Garcy AM (2015). Educational mismatch and mortality among native-born workers in Sweden A 19-year longitudinal study of 2.5 million over-educated, matched and under-educated individuals,1990-2008. Sociol Health Illn.

[15] Braig S, Peter R, Nagel G, Hermann S, Rohrmann S, Linseisen J (2011). The impact of social status inconsistency on cardiovascular risk factors, myocardial infarction and stroke in the EPIC-Heidelberg cohort. BMC Public Health.

[16] Hultin H, Lundberg M, Lundin A, Magnusson C (2016). Do overeducated individuals have increased risks of ill health?:a Swedish population-based cohort study. Sociol Health Illn.

[17] Smith BT, Smith PM, Etches J, Mustard CA (2012). Overqualification and risk of all-cause and cardiovascular mortality:evidence from the Canadian Census Mortality Follow-up Study (1991-2001). Can J Public Health.

[18] McEwen BS (1998). Protective and damaging effects of stress mediators. N Engl J Med.

[19] Frank K, Hou F (2018). Over-education and well-being:how does education-occupation mismatch affect the life satisfaction of university-educated immigrant and non-immigrant workers?. Ethn Health.

[20] World Health Organization International Statistical Classification of Diseases and Related Health Problems, 10th revision (ICD 10):WHO.

[21] Andersson T, Alfredsson L, Källberg H, Zdravkovic S, Ahlbom A (2005). Calculating measures of biological interaction. Eur J Epidemiol.

[22] Hosmer DW, Lemeshow S (1992). Confidence interval estimation of interaction. Epidemiology.

[23] Statistics Sweden (2019). Arbetsmarknaden 2018 för högutbildade utrikes födda [The labor market in 2018 for foreign born persons with a higher education] (In Swedish).

[24] Siegrist J (1996). Adverse health effects of high-effort/low-reward conditions. J Occup Health Psychol.

[25] Jalasto J, Lassmann-Klee P, Schyllert C, Luukkonen R, Meren M, Larsson M (2022). Occupation, socioeconomic status and chronic obstructive respiratory diseases - The EpiLung study in Finland, Estonia and Sweden. Respir Med.

[26] Torén K (2019). Viktigt att överväga yrke som orsak till lungsjukdom [Occupational exposures should be considered in all patients with non-malignant respiratory diseases] [In Swedish] [FTHA. Lakartidningen.

[27] Statens beredning för medicinsk och social utvärdering (SBU) (2015). Arbetsmiljöns betydelse för hjärt-kärlsjukdom:en systematisk litteraturöversikt [Occupational Exposures and Cardiovascular Disease] (In Swedish).

[28] Statens beredning för medicinsk utvärdering (SBU) (2014). Arbetsmiljöns betydelse för ryggproblem:en systematisk litteraturöversikt [Occupational Exposure and Back Disorders] (In Swedish).

[29] Statens beredning för medicinsk och social utvärdering (SBU) (2016). Arbetsmiljöns betydelse för artrosbesvär:en systematisk översikt och utvärdering av medicinska, sociala och etiska aspekter [Occupational Exposure and Osteoarthritis] (In Swedish).

[30] van der Molen HF, Foresti C, Daams JG, Frings-Dresen MH, Kuijer PP (2017). Work-related risk factors for specific shoulder disorders:a systematic review and meta-analysis. Occup Environ Med.

[31] Bracke P, Pattyn E, von dem Knesebeck O (2013). Overeducation and depressive symptoms:diminishing mental health returns to education. Sociol Health Illn.

[32] Nyberg A, Johansson G, Westerlund H, Rostila M, Toivanen S (2020). Status incongruence in human service occupations and implications for mild-to-severe depressive symptoms and register-based sickness absence:A prospective cohort study. Scand J Work Environ Health.

[33] Paul K, Moser K (2009). Unemployment impairs mental health:meta-analyses. J Vocat Behav.

[34] Helgesson M, Johansson B, Nordqvist T, Lundberg I, Vingård E (2013). Unemployment at a young age and later sickness absence, disability pension and death in native Swedes and immigrants. Eur J Public Health.

[35] Crollard A, de Castro AB, Tsai JH (2012). Occupational trajectories and immigrant worker health. Workplace Health Saf.

[36] Joona PA, Gupta ND, Wadensjö E (2014). Overeducation among immigrants in Sweden:incidence, wage effects and state dependence. IZA Journal of Migration.

[37] Åslund O, Forslund A, Liljeberg L (2017). Labor market entry of non-labor migrants –Swedish evidence. IFAU Working paper.

[38] SOU - Swedish Government Official Reports 2017:93 Klarlagd identitet (2017). Om utlänningars rätt att vistas i Sverige, inre utlänningskontroller och missbruk av identitetshandlingar. [Verified identity. About the right of foreign nationals to stay in Sweden, internal checks on foreigners, and the misuse of identity documents. enquiry emphasized the importance of verifying a person's identity] (In Swedish). Stockholm.

